# Ets-2 and C/EBP-beta are important mediators of ovine trophoblast Kunitz domain protein-1 gene expression in trophoblast

**DOI:** 10.1186/1471-2199-8-14

**Published:** 2007-02-27

**Authors:** Anindita Chakrabarty, R Michael Roberts

**Affiliations:** 1 Departments of Veterinary Pathobiology, University of Missouri-Columbia, Columbia, MO 65211, USA; 2 Department of Animal Sciences, University of Missouri-Columbia, Columbia, MO 65211, USA; 3 Department of Biochemistry, University of Missouri-Columbia, Columbia, MO 65211, USA; 4 Department of Pediatrics, Vanderbilt University Medical Center, Nashville, TN 37232, USA

## Abstract

**Background:**

The trophoblast Kunitz domain proteins (TKDPs) constitute a highly expressed, placenta-specific, multigene family restricted to ruminant ungulates and characterized by a C-terminal "Kunitz" domain, preceded by one or more unique N-terminal domains. TKDP-1 shares an almost identical expression pattern with interferon-tau, the "maternal recognition of pregnancy protein" in ruminants. Our goal here has been to determine whether the ovine (ov) *Tkdp-1 *and *IFNT *genes possess a similar transcriptional code.

**Results:**

The *ovTkdp-1 *promoter has been cloned and characterized. As with the *IFNT *promoter, the *Tkdp-1 *promoter is responsive to Ets-2, and promoter-driven reporter activity can be increased over 700-fold in response to over-expression of Ets-2 and a constitutively active form of protein Kinase A (PKA). Unexpectedly, the promoter element of *Tkdp-1 *responsible for this up-regulation, unlike that of the *IFNT*, does not bind Ets-2. However, mutation of a CCAAT/enhancer binding element within this control region not only reduced basal transcriptional activity, but prevented Ets-2 as well as cyclic adenosine 5'-monophosphate (cAMP)/PKA and Ras/mitogen-activated protein kinase (MAPK) responsiveness. *In vitro *binding experiments and *in vivo *protein-protein interaction assays implicated CCAAT/enhancer binding protein-beta (C/EBP-β) as involved in up-regulating the *Tkdp-1 *promoter activity. A combination of Ets-2 and C/EBP-β can up-regulate expression of the minimal *Tkdp-1 *promoter as much as 930-fold in presence of a cAMP analog. An AP-1-like element adjacent to the CCAAT enhancer, which binds Jun family members, is required for basal and cAMP/ C/EBP-β-dependent activation of the gene, but not for Ets-2-dependent activity.

**Conclusion:**

This paper demonstrates how Ets-2, a key transcription factor for trophoblast differentiation and function, can control expression of two genes (*Tkdp-1 *and *IFNT*) having similar spatial and temporal expression patterns via very different mechanisms.

## Background

The trophoblast Kunitz domain proteins are a family of closely related gene products uniquely expressed by trophoblast of ruminant ungulates [1,2]. Each protein is characterized by the presence of a well-conserved, carboxyl-terminal ~65 amino acid domain, typically found in the Kunitz family of serine peptidase inhibitors. The Kunitz domain is an evolutionary conserved module, existing in *C. elegans*, *D. melanogaster*, and all known vertebrates, that generally functions as a serine peptidase inhibitor, although functions unrelated to peptidase inhibition have also been documented [3-6]. This independently folding unit is preceded by one or more "repeat" sequences of ~80 amino acids of unknown function, which have similar but not identical sequences [1,2]. TKDPs represent one of the most abundant classes of secretory proteins produced by the conceptus during the peri-implantation period and throughout gestation [1,2,7,8]. Although the function of these proteins remains unclear, their unusually high expression at the interface between the maternal and fetal systems and the presence of conserved Kunitz units at their carboxyl termini strongly suggest an important role during pregnancy that relates to the evolution of the non-invasive placental type encountered in ruminant species.

Although expressed abundantly, the concentrations of different TKDP mRNAs do not remain constant over the course of pregnancy. Instead, expression of each TKDP appears to be quite tightly regulated and confined to specific stages of fetal development. For example, ovTKDP-1 and its bovine ortholog (boTKDP-1) are expressed significantly for just a few days preceding the period when the trophoblast begins to adhere tightly to the uterine wall [2] In this respect, the temporal expression pattern of TKDP-1 and that of IFN-τ, the major signal for maternal recognition of pregnancy in cattle and sheep are remarkably similar. For example, both TKDP-1 and IFN-t are secreted by the trophoblast mononuclear cells during days 13–21 of pregnancy in sheep [7]. In view of the physiological importance of IFN-τ in ensuring the survival of the conceptus [9,10] and the fact that both TKDP-1 and IFN-τ are up-regulated massively and simultaneously at a particular critical time when many embryos are lost, it seems likely that ovTKDP-1 has some function important for mediating maternal-conceptus interaction during the peri-attachment/implantation period of conceptus development.

The strikingly similar spatial-temporal expression pattern of TKDP-1 and IFN-τ raises the possibility that these genes (*Tkdp-1 and IFNT*) representing these proteins are controlled by very similar transcriptional mechanisms and possibly share common transcription factor binding elements in their promoter regions. The major focus of the current research has been to gain insight into the control of *Tkdp-1 *gene expression and to determine whether the mechanisms involved resembled those associated with the regulation of the *IFNT*. One important goal has been to elucidate whether or not there is a common theme to the transcriptional regulation of genes that are first expressed as the trophoblast begins to differentiate functionally early in pregnancy and secrete its signature gene products. The outcome of this research could, therefore, have implications to the establishment and/or maintenance of pregnancy and the specification of trophoblast function in all mammals.

The data presented here demonstrates Ets-2 having an important role in activating the transcription of the *ovTkdp-1 *gene in *in vitro *cell culture systems. However, unlike the *IFNT *promoter, which directly interacts with Ets-2 via consensus Ets-binding site, the Ets-2 effect on the *Tkdp-1 *promoter is indirect and mediated by protein-protein interaction with C/EBP-β. In other words, Ets-2-dependent transactivation of genes with nearly identical spatial and temporal expression patterns can be mediated by different mechanisms.

## Results

### Expression patterns of the ovTKDP-1 and IFN-τ messages during pregnancy

**Figure 1:**
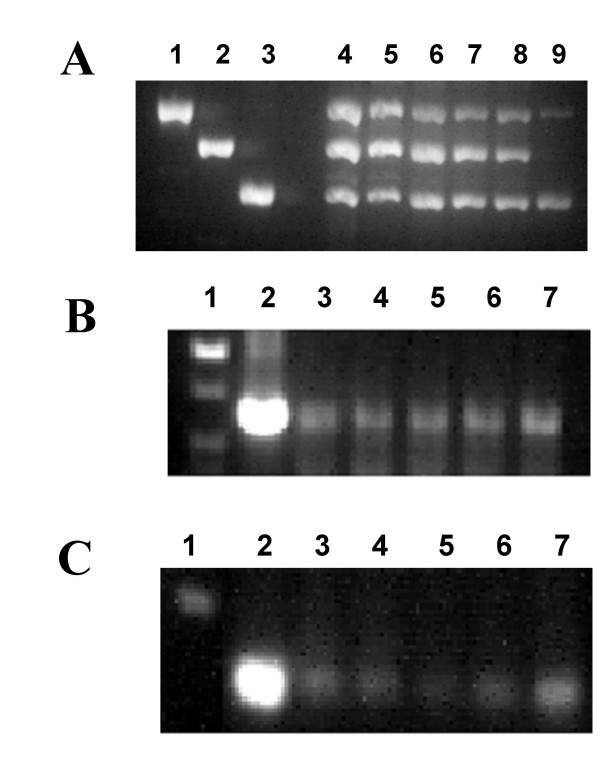
**Detection of ovTKDP-1, IFN-τ, Ets-2 and C/EBP-β mRNA in sheep trophectoderm**. RT reactions were performed on sheep trophoblast RNA collected from different days of pregnancy. After binding to oligo dT primer, reverse transcription and PCR amplification with primers specific for each message were performed. **A) **Comparison of ovTKDP-1 and IFN-τ expression during the peri-implantation period of conceptus development. Lanes 1–3: positive control PCR with TKDP-1, IFN-τ and S25 plasmid templates, respectively; lanes 4–9: PCR with days 14, 15, 16, 17, 19 and 25 sheep trophoblast cDNA. **B) **Ets-2 mRNA expression during the peri-implantation period of conceptus development. Lane 1: DNA marker; lane 2: positive control PCR with Ets-2 expression plasmid; lanes 3–8: PCR with day 14, 15, 16, 17 and 25 cDNA. **C) **C/EBP-β mRNA expression;. Lane 1: DNA marker; lane 2: positive control PCR with C/EBPβ expression plasmid; lanes 3–8: PCR with day 14, 15, 16, 17 and 25 cDNA.

Although TKDP-1 and IFN-τ mRNA are expressed within a comparable window of early pregnancy [7], side by side comparisons have not been made. We therefore conducted RT-PCR with equal quantities of RNA extracted from sheep conceptuses collected at different days of early pregnancy. The expression patterns for ovTKDP-1 and ovIFN-τ mRNAs were similar both in magnitude and over developmental time (Fig. 1A). Both transcripts were detectable in sheep conceptuses at day 14 of pregnancy, with concentrations peaking at day 15 and subsequently declining after day 17 to become almost undetectable by day 25 relative to the amplified product derived from the endogenous control, ribosomal protein S25.

### Constitutive transcriptional activities of the truncated promoter-Luciferase (Luc) reporter constructs from the Tkdp-1 gene in human choriocarcinoma JEG-3 cells

**Figure 2:**
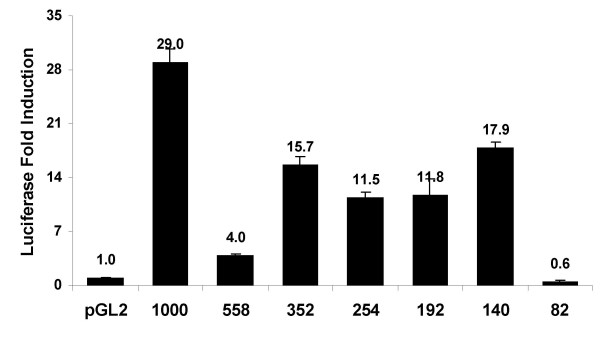
**Expression of a series of *Tkdp-1 *promoter-*Luc *reporter constructs transfected into JEG-3 cells**. *Luc *activities are expressed relative to that of the promoter-less pGL2-Basic *Luc *reporter construct, with data shown as means ± SEM (n = 3; P < 0.05: pGL2 vs. 1000, 352, 254, 192, 140).

Different length fragments from 1 Kbp region upstream of the transcription start point (*tsp*) of the *Tkdp-1 *gene were used to generate seven sequentially 5^/^-truncated *Tkdp-1 *promoter-*Luc *reporter constructs (1000, 558, 352, 254, 192, 140 and 82 bp from the *tsp*). These constructs were transiently transfected into JEG-3 cells and their expression compared relative to the *Luc *activity from the promoter-less pGL2 Basic vector (Fig. 2). All but the 82 bp promoter-reporter constructs were able to drive *Luc *reporter gene significantly better than the promoter-less reporter construct. The 1000 bp construct had the maximum basal promoter activity, and this activity was reduced significantly when the promoter was shortened to 558 bp. Promoter activity increased again in the 352, 254, 192 and 140 bp constructs, but was never completely restored to that of the 1000 bp construct. These data suggest that there are two enhancer/activator elements present, one distal, between -558 and -1000 bp, and the other more proximal, lying between 140 and 82 bp. There may also be a cryptic repressor element lying between the 558 bp and 352 bp promoter fragments, although another explanation for the poor activity of the 558 construct is that it folds in such a way that it is less easily transcribed than its longer and shorter relatives. Since the 140 bp construct was the shortest one able to drive *Luc *expression effectively, contained the proximal enhancer element, and included several relevant transcription factor binding sites (see below) our efforts became focused on this control region of the gene.

### Putative transcription factor-binding sites on theTkdp-1 promoter

**Figure 3:**
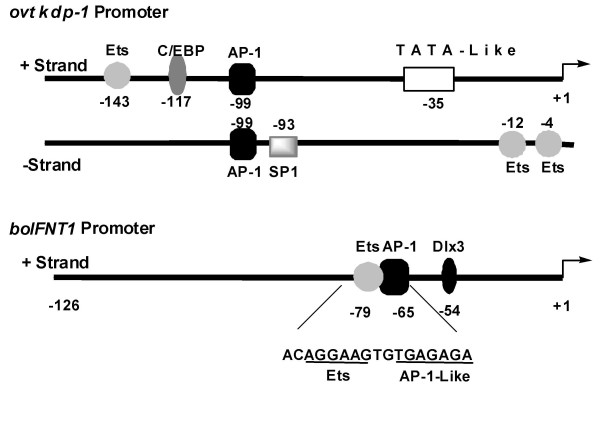
**Possible transcription factor-binding sites in the proximal *ovTkdp-1 *and *boIFNT1 *promoters**. The sequence of the Ets-AP-1 composite enhancer in the *boIFNT1 *promoter is also shown [23]. The *tsp *is designated +1.

Fig. 3 illustrates putative transcription factor binding sites within 140 bp upstream of the *tsp *of the *Tkdp-1 *promoter that can also be identified within the *boIFNT1 *proximal promoter (-126 to +50) and have been implicated in the basal expression of *IFNT *genes *in vitro *[11,12]. No Ets-2/AP-1 composite enhancer motif comparable to the one essential for *IFNT *gene transcription is detectable within the proximal 140 bp of the *Tkdp-1 *promoter, although a number of potential Ets-factor binding elements are present. None of these sites possesses a sequence identical to the Ets-2-binding element found within the *IFNT *promoter (-79 ACAGGAAGTG -70), although each does possess the central GGA motif that is an invariable feature of all Ets-factor-binding sites [13,14] (Fig. 3 and additional data file 1 – Fig. 1). A possible AP-1 element is present in the *Tkdp-1 *promoter, but its sequence is different from that of the one placed just upstream of the Ets-2-binding site on all known *IFNT *genes (TGAGAGA versus TGAG/CTCA) [11].

### Effects of over-expressing Ets family transcription factors on the transcriptional activity of the Tkdp-1 promoter in JEG-3 cells

**Figure 4:**
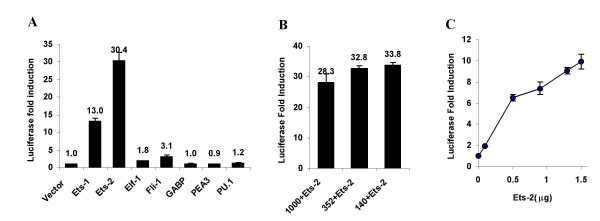
**Transactivation of the *Tkdp-1 *promoter by Ets-2**. **A) **Effects of different Ets family transcription factors on reporter expression from the 1000 bp *Tkdp-1 *promoter-*Luc *reporter construct in JEG-3 cells. *Luc *activities (means ± SEM; n = 3) are expressed relative to that of the basal activity of the 1000 bp construct. Following values differ significantly (p < 0.05): 1000 vs. 1000+Ets-1 and Ets-2; 1000+Ets-1 vs. 1000+Ets-2. **B) **Effect of Ets-2 on the transcriptional activities of three 5^/^-truncated *Tkdp-1 *promoter-*Luc *reporter constructs 1000, 352 and 140 in JEG-3 cells. *Luc *activities are expressed relative to that of the basal activity of each of the truncated constructs and data are shown as mean ± SEM (n = 3). For each truncated construct, the fold-induction in reporter activity after co-transfection with Ets-2 is significantly different from that without Ets-2 (P < 0.05). **C) **Effect of increasing concentrations of Ets-2 on the activity of the 1000 bp *Tkdp-1 *promoter-*Luc *reporter construct. *Luc *activities are expressed relative to that of the basal activity of the 1000 bp construct and data are shown as mean ± SEM (n = 3). Following values differ significantly (p < 0.05): 1000 vs. 0.5 μg, 0.9 μg, 1.3 μg and 1.5 μg Ets-2.

Although expressed widely in adult and embryonic tissues, the Ets family transcription factor Ets-2 is crucial for placental development [15]. It is also present in ovine conceptus tissue at the time that IFN-τ is produced and is known to support expression of several genes that are restricted to trophoblast [16-22], including *IFNT *[23]. Since the *Tkdp-1 *gene possesses several Ets-like sites within its promoter region, transient transfection experiments were performed to test the effects of over-expressing several Ets family members on the transcriptional activity of the 1000 bp promoter-reporter construct in JEG-3 cells (Fig. 4A). In order to provide roughly comparable expression, each of these Ets expression constructs was driven by the same cytomegalovirus promoter, although protein stability and other variables could not be controlled. Among six different Ets-related transcription factors examined (Ets-1, Ets-2, Elf-1, Fli-1, PEA3, PU.1 and GABP-α,-β) only Ets-1 and Ets-2 had significant ability to up-regulate the transcriptional activity of the 1000 bp *Tkdp-1 *promoter. Of the two, Ets-2 was clearly the superior (30-fold versus 13-fold).

To examine which part of the *Tkdp-1 *promoter is responsive to Ets-2, an equal amount of three truncated *Tkdp-1 *promoter-reporter constructs, 1000 bp, 352 bp and 140 bp, was co-transfected into JEG-3 cells with the Ets-2 expression plasmid. Ets-2 over-expression increased *Luc *activity of all three constructs quite similarly (Fig. 4B), although the 140 bp promoter was slightly more responsive than the 1000 bp promoter (Fig. 4B: 28-fold for 1000 versus 34-fold for 140). These data suggested that the region of the promoter targeted by Ets-2 is within 140 bp of the *tsp*. We also tested the effect of Ets-2 over-expression on the transcriptional activity of the promoter-less *Luc *reporter vector (pGL-2 Basic) (data not shown). As expected, Ets-2 had no significant effect on the reporter expression in absence of a promoter, indicating that the Ets-2-mediated activation of the *Tkdp-1 *promoter-*Luc *construct is a promoter-specific effect.

Ets-2-mediated activation of the *Tkdp-1 *promoter was dose-dependent (Fig. 4C). Differences in fold-activation between experiments probably reflect the quality of the plasmid DNA and other variables not easily controlled in transfection studies. A time-course experiment was also performed as a preliminary test to determine whether the Ets-2-responsiveness of the *ovTkdp-1 *promoter is early or delayed. An early response is indicative of a direct effect while a delayed response would suggest Ets-2-mediated up-regulation of some other transcription factor necessary for expression of the *Tkdp-1 *gene. When JEG-3 cells, co-transfected with Ets-2 and the 1000 bp *ovTkdp-1 *promoter constructs, were harvested at various time points after transfection (data not shown), Ets-2-responsiveness of the 1000 bp construct was clearly evident within 6 h of transfection, suggesting that Ets-2 directly activates the *Tkdp-1 *promoter.

### Ras/MAPK-mediated activation of theTkdp-1 promoter in 3T3 cells

**Figure 5:**
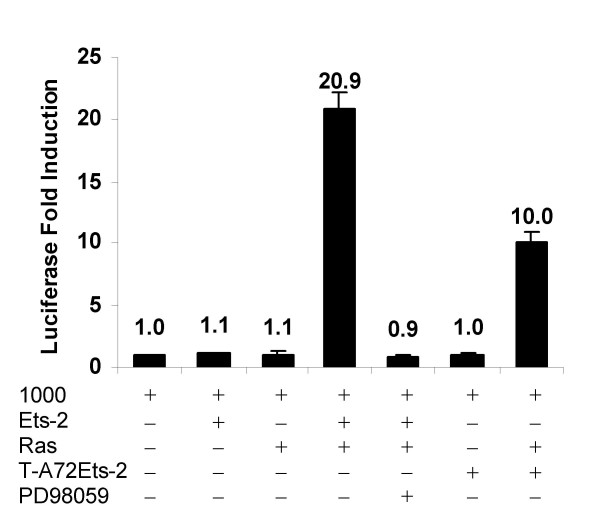
**Effects of Ets-2 and activated Ras on the *Tkdp-1 *1000 bp promoter activity in NIH3T3 cells**. *Luc *activities are expressed relative to that of the basal activity of the 1000 bp construct. Data are shown as means ± SEM (n = 3). The following values differ significantly (p < 0.05): 1000 vs. Ets-2+Ras and T-A72Ets-2+Ras; Ets-2+Ras vs. T-A72Ets-2+Ras.

Ets-2 and the related transcription factor Ets-1 bind to specific DNA elements via their Ets domains, but often require activation through Ras/MAPK-dependent phosphorylation at specific residues in order to participate effectively in target gene transcription [11,16,24-28]. To test whether the 1000 bp *Tkdp-1 *promoter-reporter construct was similarly responsive to MAPK activation, it was co-transfected with a constitutively active Ras expression plasmid in presence and absence of Ets-2 in mouse fibroblast 3T3 cells. These mouse fibroblasts were used because, unlike choriocarcinoma cells, they possess low endogenous levels of Ets-2 and activated Ras [11], so that the effect of over-expression is easily demonstrable. Ras in combination with Ets-2, but not by itself, caused a 21-fold activation of the 1000 bp promoter construct (Fig. 5). This stimulation of *Tkdp-1 *promoter activity was reduced approximately 96% by treating the transfected cells with the MAPK kinase (MEK1) inhibitor PD98059. If the codon encoding the Ras/MAPK target site, Thr 72, was mutated, *Tkdp-1 *promoter activity was reduced by about 50% compared to that obtained with the Ras and wild type Ets-2 combination. These data show that the *Tkdp-1 *promoter can be up-regulated by the Ras/MAPK pathway acting through Ets-2.

### cAMP/PKA-mediated activation of the Tkdp-1 promoter in JEG-3 cells

The cAMP/PKA signaling pathway has been shown to modulate the Ets-dependent activation of several trophoblast-specific genes, including the human chorionic gonadotropin (*hCG)-α *and *β *subunit genes [16,17] and *IFNT *[17]. Treatment of JEG-3 cells with increasing concentrations of the cAMP analog, 8-Br-cAMP, after co-transfecting the cells with the 1000 bp *Tkdp-1 *promoter-reporter construct and the Ets-2 expression plasmid provided only a 2–3-fold enhancement of the Ets-2 effect (Additional data file 1 – Fig. 2). 8-Br-cAMP alone, i.e. in absence of over-expressed Ets-2, had no significant ability to up-regulate the promoter (data not shown).

**Figure 6:**
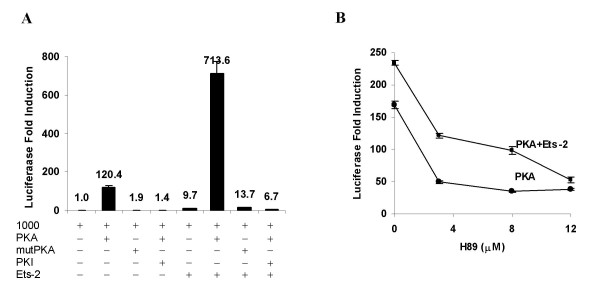
**Effects of Ets-2 and activated PKA on the *Tkdp-1 *1000 bp promoter activity in JEG-3 cells**. Expression of the 1000 bp promoter-*Luc *construct **A) **following co-transfection with activated PKA, activated PKA+PKI, mutPKA, Ets-2, Ets-2+activated PKA, Ets-2+activated PKA+PKI, Ets-2+mutPKA and **B) **in cells exposed to the PKA inhibitor H89 in presence and absence of co-transfected Ets-2. *Luc *activities are expressed relative to that of the basal activity of the 1000 bp construct and data are shown as mean ± SEM (n = 3). The following values are significantly different at P < 0.05: **A) **1000 vs. 1000+PKA, 1000+Ets-2, 1000+Ets-2+PKA; 1000+PKA vs. 1000+mutPKA, 1000+PKA+PKI; 1000+Ets-2+PKA vs. 1000+Ets-2+mutPKA, 1000+Ets-2+PKA+PKI and **B) **1000+PKA vs. 1000+PKA+3 μMH89, 1000+PKA+8 μMH89; 1000+PKA+12 μM H89; 1000+Ets-2+PKA vs. 1000+Ets-2+PKA+3 μM H89, 1000+Ets-2+PKA+8 μM H89, 1000+Ets-2+PKA+12 μM H89

One possibility of explaining why 8-Br-cAMP had such a minor effect on the *Tkdp-1 *promoter was that in JEG-3 cells PKA, the downstream target of cAMP, is present in limiting amounts. Accordingly, the effects of a constitutively active catalytic subunit of PKA were examined. As shown in Fig. 6A, over-expression of the constitutively active catalytic subunit of PKA led to a 120-fold up-regulation of the basal activity of the 1000 bp *Tkdp-1 *promoter in JEG-3 cells. When Ets-2 was also over-expressed, activity of the promoter was enhanced over 700-fold. The PKA and PKA+Ets-2 effects on the *Tkdp-1 *promoter activation were inhibited over 95% by expression of a PKA inhibitor (PKI) construct (Fig. 6A). Additionally, substitution of the plasmid expressing the active form of PKA with one with a mutated catalytic site (mutPKA) failed to up-regulate the *Tkdp-1 *promoter (Fig. 6A). Finally, treatment of transfected cells with a pharmacological PKA inhibitor, H89, caused dramatic reduction in promoter activity, both in absence and presence of Ets-2, in a concentration-dependent manner (Fig. 6B). These data indicate that the *Tkdp-1 *promoter is responsive to the cAMP/PKA signal transduction pathway and that Ets-2 acts synergistically with this pathway to up-regulate this promoter.

### Indirect interaction between Ets-2 and the Tkdp-1 promoter

**Figure 7:**
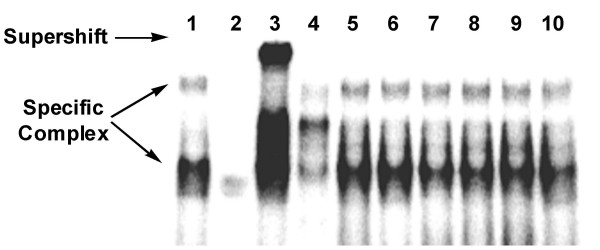
**Competition EMSA with recombinant Ets-2 protein and a ^32^P-labeled ds Ets-2 consensus oligonucleotide**. Lane 1: labeled oligonucleotide+Ets-2-GST-fusion protein; lane 2: labeled oligonucleotide+GST protein; lane 3: labeled oligonucleotide+Ets-2-GST-fusion protein+anti-Ets-2 IgG; lane 4: labeled oligonucleotide+Ets-2-GST-fusion protein+unlabeled Ets-2 consensus oligonucleotide (250-fold molar excess); lanes 5–10: labeled oligonucleotide+Ets-2-GST-fusion protein+unlabeled overlapping oligonucleotides from the *Tkdp-1 *promoter (-150 to +20) at 250-fold molar excess.

As mentioned previously, other than having the core GGA sequence, none of the putative Ets-like sites within the *Tkdp-1 *promoter showed a close match with either the consensus Ets-2-binding motif (C/A)(C/A)GGA(A/T)(A/G) or the well defined, functional site (CAGGAAG) in the *IFNT *promoter. In order to determine whether any of these putative Ets-like sites within the *Tkdp-1 *promoter binds Ets-2, competition electrophoretic mobility shift assay (EMSA) was performed. The 140 bp minimal promoter was chosen because it had multiple potential Ets-like sites (Fig. 3) and provided full Ets-2 responsiveness (Fig. 4B). Initial experiments in which we employed EMSA to demonstrate an interaction between recombinant Ets-2 and oligonucleotides representing these potential Ets-binding sequences failed to reveal the formation of any complexes (data not shown). As shown in Fig. 7, none of the six potential competitor oligonucleotides (lanes 5–10) was able to disrupt either the slower or faster migrating DNA-protein complexes formed between the ^32^P-labeled Ets consensus oligonucleotide and ovEts-2-GST-fusion protein (lanes 1 and 3). The slight variation in intensities in the faster and slower migrating specific complexes across lanes 5–10 likely resulted from differences in gel loading. EMSA experiments employing labeled oligonucleotides representing the putative Ets-binding sites within the 140 bp promoter failed to provide specific complexes with recombinant Ets-2 (data not shown). Together, these results indicate that Ets-2 is unable to associate with the proximal promoter and that none of the putative Ets-binding sites present is likely to be functional.

### Activation of the Tkdp-1 promoter by C/EBPs in JEG-3 cells

**Figure 8:**
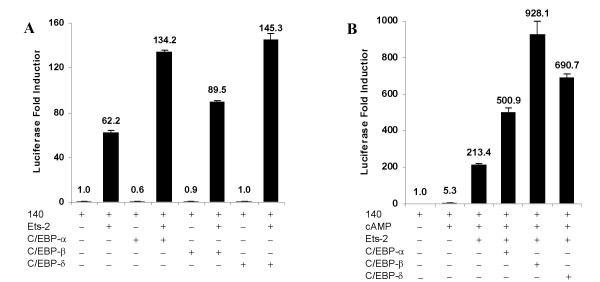
**Effects of C/EBP-α, -β and -δ on the *Tkdp-1 *140 bp promoter activity in JEG-3 cells**. *Luc *expression driven by the minimal promoter in cells co-transfected with each of the C/EBP isoforms **A) **in the absence and presence of over-expressed Ets-2 and **B) **in the presence of over-expressed Ets-2 and 250 μM 8-BrcAMP. *Luc *activities are expressed relative to that of the basal activity of the 140 bp promoter and data are shown as mean ± SEM (n = 3). The following values are significantly different at P < 0.05: **A) **140 vs. Ets-2, C/EBP-α+Ets-2, C/EB-Pβ+Ets-2, C/EBP-δ+Ets-2 and **B) **140 vs. cAMP+Ets-2, cAMP+Ets-2+C/EBP-α, cAMP+Ets-2+C/EBP-β, cAMP+Ets-2+C/EBP-δ; cAMP+Ets-2 vs. cAMP+Ets-2+C/EBP-β, cAMP+Ets-2+C/EBP-δ. cAMP+Ets-2+C/EBP-β is significantly different (P < 0.05) from both cAMP+Ets-2+C/EBP-α and cAMP+ Ets-2+C/EBP-δ.

C/EBPs are basic-leucine zipper transcription factors, comprising of six family members (-α, -β, -γ, -δ, -ε and -ξ) [29]. C/EBP proteins are known to interact with Ets family members [30-33]. The 140 bp *Tkdp-1 *promoter possesses a consensus CCAAT/enhancer element (TTATGCAAT) [34] located between -117 and -109 bp (Fig. 3). Accordingly, determined whether the 140 bp *Tkdp-1 *promoter construct was responsive to over-expression of three C/EBP family members (-α, -β and -δ) (Fig. 8A). None of the three C/EBP family members used here had an effect on the basal promoter activity, and only a slight increase was observed in presence of Ets-2. When Ets-2 was over-expressed in presence of 250 μM 8-Br-cAMP (Fig. 8B) each family member further up-regulated the *Tkdp-1 *promoter activity, with C/EBP-β being the most potent. Together, these results suggest that the Ets-2/cAMP/PKA effect on the promoter noted in Fig. 6 is possibly mediated through C/EBP-β.

### Mutation of the C/EBP binding site in theTkdp-1 minimal promoter reduces its activity

**Figure 9:**
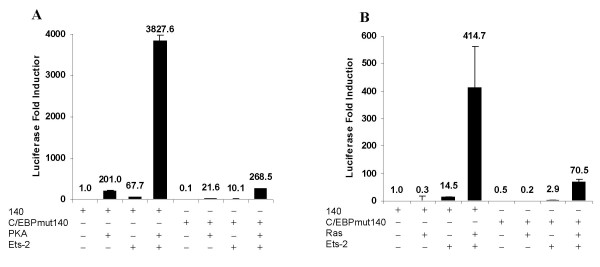
**Effects of the CCAAT/enhancer element (-117 to -109) mutation on the *Tkdp-1 *140 bp promoter activity in A) JEG-3 and B) NIH3T3 cells**. **A) **Basal, activated PKA, Ets-2, Ets-2+activated PKA-dependent activation of the wild type and C/EBP-mutated minimal promoter in JEG-3 cells. **B) **Basal, activated Ras, Ets-2, Ets-2+activated Ras-dependent activation of the wild type and C/EBP-mutated promoter in NIH3T3 cells. *Luc *activities are expressed relative to that of the basal activity of the 140 bp promoter and data are shown as mean ± SEM (n = 3). Following values are significantly different at P < 0.05: **A) **140+PKA vs. C/EBPmut140+PKA; 140+Ets-2 vs. C/EBPmut140+Ets-2; 140+Ets-2+PKA vs. C/EBPmut140+Ets-2+PKA and **B) **140+Ets-2 vs. C/EBPmut140+Ets-2 and 140+Ets-2+Ras vs. C/EBPmut140+Ets-2+Ras.

When the CCAAT/enhancer element (TTATGCAAT) of the 140 bp *Tkdp-1 *promoter-reporter construct was mutated (TTATcCccc) both the basal transcriptional activity and the effects of Ets-2, PKA and Ets-2+PKA were markedly reduced (~90 %) compared to the wild type promoter in JEG-3 cells (Fig. 9A). That the C/EBP-binding site mutation had no additional effect on Ets-2-dependent than on basal promoter activation was somewhat surprising, However, basal promoter activity is quite high in JEG-3 cells, e.g. compared to the non-trophobast cell line, NIH3T3, possibly because of the higher content of endogenous Ets2 and other essential transcription factors present in these trophoblast cells. Accordingly, the effect of the mutation on basal activity may be difficult to distinguish from the effect observed when Ets-2 is expressed ectopically. Reduction in the basal promoter activity and the Ets-2 or Ets-2+Ras responsiveness was also observed in the 3T3 mouse fibroblast cell line when the CCAAT/enhancer element was mutated (Fig. 9B), and, the effect of the mutation when Ets-2 was over-expressed was more pronounced. Thus, mutation of the CCAAT/enhancer element largely eliminated the promoter's ability to respond to Ets-2 and the cAMP/PKA or the Ras/MAPK signal transduction pathways. These data along with our earlier observation (Fig. 8A) that C/EBP-α, -β or -δ has no effect on the reporter gene expression by its own, suggest that the effects of PKA, Ets-2, Ets-2+PKA and Ets-2+Ras on the 140 bp *Tkdp-1 *promoter are mediated through the C/EBP-binding site, conceivably through a direct interaction of Ets-2 with C/EBP-β.

### C/EBP-β directly interacts with theTkdp-1 promoter

**Figure 10:**
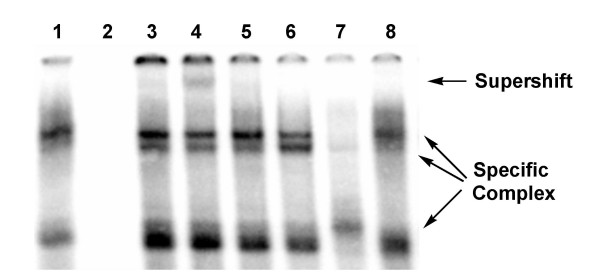
**EMSA with ^32^P-labeled C/EBP oligonucleotide (-117 to -109) and JEG-3 cell extracts (CE)**. Lane 1: labeled oligonucleotide+CE; lane 2: labeled oligonucleotide-CE; lane 3: labeled oligonucleotide+CE+C/EBP-α IgG; lane 4: labeled oligonucleotide+CE+C/EBP-β IgG; lane 5: labeled oligonucleotide+CE+C/EBP-δ IgG; lane 6: labeled oligonucleotide+CE+non-immune rabbit IgG; lanes 7–8: labeled oligonucleotide+CE+250-fold molar excess of unlabeled wild type (lane 7) and mutated (lane 8) C/EBP competitor oligonucleotides.

In order to identify which of the three C/EBP family members used in the transfection experiments (Fig. 8A and 8B) interacts with the 140 bp promoter *in vitro*, we performed EMSA with a ^32^P-labeled double-stranded (ds) oligonucleotide possessing the C/EBP-binding site of the *Tkdp-1 *promoter, JEG-3 cell extracts, and antibodies specific for C/EBP-α, -β, and -δ (Fig. 10). As shown in lane 1, a specific DNA-protein complex was formed between the CCAAT/enhancer element and JEG-3 cell extracts. Excess unlabeled competitor oligonucleotide containing the wild-type C/EBP-binding site (TTATGCAAT; lane 7) inhibited formation of the specific complex when it was added to the reaction mixture. However, an unlabeled oligonucleotide possessing a mutated C/EBP-binding site (TTATcCccc; lane 8) failed to compete in the binding reaction. The addition of an antiserum directed against individual C/EBPs only provided a major "supershift" when the anti-C/EBP-β reagent was employed (lane 4). There was a minor effect noted with anti-C/EBP-α (lane 3). Neither anti-C/EBP-δ (lane 5) nor non-immune rabbit IgG (lane 6) had any effect. Thus C/EBP-β would appear to be the major isoform of C/EBP that binds the TTATGCAAT element present in the 140 bp minimal promoter of the *Tkdp-1 *gene.

### Interaction between C/EBP-β and Ets-2 in JEG-3 cells

**Figure 11:**
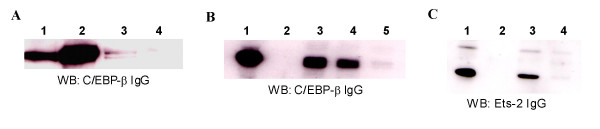
**C/EBP-β and Ets-2 interaction demonstrated by A) co-immunoprecipitation, B) and C) pull-down assays with biotinylated C/EBP oligonucleotide (-117 to -109) in JEG-3 cell extracts (CE)**. **A) **Lane 1: JEG-3 CE (positive control for western blot); lane 2: immunoprecipitation (IP) with C/EBP-β IgG (positive control for IP); lane 3: IP with Ets-2 IgG; lane 4: IP with non-immune rabbit IgG (negative control for IP). Western blot (WB) was performed with C/EBP-β IgG. **B) **WB performed with C/EBP-β IgG as detection reagent following pull-down assays. Lane 1: JEG-3 CE (positive control for WB); lane 2: biotinylated C/EBP oligonucleotide; lane 3: JEG-3 CE+biotinylated C/EBP oligonucleotide; lane 4: JEG-3 CE+biotinylated C/EBP oligonucleotide+250-fold molar excess unlabeled mutated C/EBP oligonucleotide; lane 5: JEG-3 CE+biotinylated C/EBP oligonucleotide+250-fold molar excess unlabeled wild type C/EBP oligonucleotide. **C) **WB with Ets-2 IgG as the detection agent following pull-down assays. Lane 1: JEG-3 CE (positive control for WB); lane 2: biotinylated C/EBP oligonucleotide; lane 3: JEG-3 CE+biotinylated C/EBP oligonucleotide; lane 4: JEG-3 CE+biotinylated C/EBP oligonucleotide+250-fold molar excess unlabeled wild type C/EBP oligonucleotide.

Co-immunoprecipitation (Co-IP) and oligonucleotide pull-down experiments were performed to demonstrate the *in vivo *interaction between C/EBP-β and Ets-2 in JEG-3 cells. For the former, endogenous Ets-2 in JEG-3 cell extracts was allowed to form an immune complex with a rabbit polyclonal Ets-2-specific immunoglobulin, collected on Protein-A beads and then subjected to western blot analysis with C/EBP-β-specific antiserum (Fig. 11A, lane 3). The data indicate that endogenous C/EBP-β is associated with Ets-2 in JEG-3 cell extracts. No C/EBP-β protein was detected when non-immune rabbit IgG was substituted for the anti-Ets-2 antiserum (lane 4). As positive control, endogenous C/EBP-β was collected with C/EBP-β-specific antiserum and the western blot developed with the same antiserum (lane 2).

When a streptavidin-bound, biotinylated ds oligonucleotide representing the C/EBP-binding region of the *Tkdp-1 *promoter was used as a bait to collect proteins present in JEG3 cells, the oligonucleotide was able to trap endogenous C/EBP-β (lane 3, Fig. 11B). This ability to bind C/EBP-β was inhibited in the presence of an excess of unlabeled wild type competitor oligonucleotide, but not by unlabeled mutated competitor (lanes 5 versus 4). The C/EBP-binding site containing biotinylated oligonucleotide was able to trap Ets-2 as well as C/EBP-β (lane 3, Fig. 11C), even though it did not possess a known Ets-binding sequence. The Ets-2-specific bands were not detected when excess unlabeled, wild-type competitor oligonucleotide was included in the reaction mixture (lane 4, Fig. 11C). These data provide conclusive evidence that Ets-2 associates with C/EBP-β while the latter is bound to its CCAAT-binding-sequence. The possibility that Ets-2 bound to the CCAAT/enhancer element directly is unlikely in view of earlier data (Fig. 7).

### Mutation of the AP-1-binding site does not affect the Ets-2-responsiveness of the Tkdp-1 promoter

**Figure 12:**
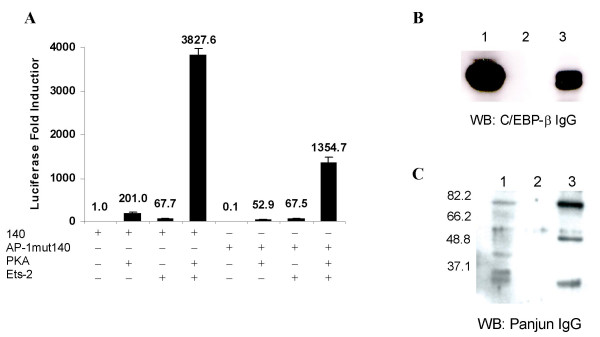
**Importance of the AP-1 element (-99 to -93) for the *Tkdp-1*.140 bp promoter activity in JEG-3 cells. A) **Effects of the AP-1 element mutation on the basal, activated PKA, Ets-2 and Ets-2+activated PKA-dependent activation of the 140 bp *Tkdp-1 *promoter-*Luc *reporter construct in JEG-3 cells. *Luc *activities are expressed relative to that of the basal activity of the 140 bp promoter and data are shown as mean ± SEM (n = 3). Following values are significantly different at P < 0.05: 140+PKA vs. AP-1mut140+PKA and 140+Ets-2+PKA vs. AP-1mut140+Ets-2+PKA. **B) **and **C) **Pull-down assays with biotinylated AP-1 oligonucleotide (-99 to -93 in the *tkdp-1 *promoter) and JEG-3 cell extracts(CE). Following pull-down a ssays, WB analysis was performed with **B) **C/EBP-β IgG and **C) **pan-Jun IgG. Lane 1: JEG-3 CE (positive control for WB); lane 2: biotinylated AP-1 oligonucleotide; lane 3: JEG-3 CE+biotinylated AP-1 oligonucleotide. The molecular weight standards are shown on the left-hand side.

The 140 bp *Tkdp-1 *promoter possesses a conserved AP-1 element (-99 to -93) immediately downstream of the CCAAT/enhancer element (-117 too -109). In order to determine the functional importance of this site for transcriptional activation of the promoter, site-directed mutagenesis followed by transient transfection experiments were performed. As demonstrated in Fig. 12A, mutation of the AP-1 element caused up to 90% reduction in basal activity and 74% reduction in PKA-dependent promoter activation. This mutation did not affect the Ets-2-mediated *ovTkdp-1 *promoter activation, although it reduced the effect of the Ets-2 plus PKA combination on the promoter by up to 65%. Together these data indicate that the AP-1 element adjacent to the C/EBP-β-binding site is important in regulating transcription from the *Tkdp-1 *minimal promoter and for activation of transcription by the cAMP/PKA signaling pathway, but is not involved in the recruitment of Ets-2 to the promoter.

### The AP-1 element in the 140 bpTkdp-1 promoter interacts with C/EBP-β

Pull-down assays were conducted in JEG-3 extracts with a biotinylated, ds oligonucleotide containing positions -99 to -93 of the *Tkdp-1 *promoter (Fig. 12B and 12C). Some interactions between this element and several Jun family transcription factors (c-Jun, Jun B, Jun D and p39) could be demonstrated by western blotting (Fig. 12C). By contrast none of the Fos-related proteins (c-Fos, Fos B, Fra-1 and Fra-2) was pulled down from the JEG-3 cell extracts (negative results not shown). Unexpectedly, this AP-1 element-protein complex contained C/EBP-β, suggesting that whatever bound to this sequence also associated with C/EBP-β (Fig. 12B).

### Expression of Ets-2 and C/EBP-β transcripts in conceptuses

RT-PCR analyses performed on RNA extracted from sheep conceptuses/trophoblast at days 14, 15, 16, 17 and 25 of pregnancy showed that C/EBP-β mRNA, like Ets-2 mRNA, is expressed relatively uniformly over these days of conceptus development (Fig. 1B and 1C). This expression pattern is in sharp contrast to that of the *Tkdp-1 *gene, whose expression pattern is maximal around day 17 and then tails off by day 25.

## Discussion

TKDP-1 is an abundant secretory product of the ruminant placental trophoblast cells during the peri-implantation stage of pregnancy, a period when the enlarging blastocyst starts to signal its presence to the mother in preparation for establishing an intimate contact with the uterine endometrium [2,7,35]. This stage of conceptus development is also marked by the production of IFN-τ, the protein responsible for "rescue" of the corpus luteum during early pregnancy in ruminant ungulate species, such as cattle, sheep, goat and deer. Like ovTKDP-1, IFN-τ is a product of trophoblast mononuclear cells, and its production rises sharply as the blastocyst expands and begins to elongate, presumably in response to growth factors, hormones and cytokines present in the uterine secretions of the mother [2,11,12]. Many of such factors operate through activation of established signal transduction pathways, including the Ras/MAPK and cAMP/PKA pathways [12,36,37]. Shutdown of expression after the trophoblast begins to adhere to the uterine wall might be due to lack of access of the adherent trophectoderm to these maternal factors, which probably originate from the uterine glands of the underlying endometrium. The striking similarity in the expression patterns of ovTKDP-1 and IFN-τ, which is illustrated in Fig. 1A, led us to speculate that the genes for these two proteins might be controlled by similar transcriptional mechanisms, involving the same transcription factors and signal transduction pathways.

The starting point for our study on *Tkdp-1 *transcriptional control was the Ets family transcription factor, Ets-2, a homolog for the viral oncogene of the avian leukemia virus E26 [38]. Although expressed widely in adult and embryonic tissues, Ets-2 is required for placental development in the mouse [39] and supports expression of several genes, including the *IFNT*, whose expression provide a phenotypic "signature" for trophectoderm [15-21,23]. The promoters of each of these genes possess functional Ets-2-binding sites which, when mutated, cause almost complete loss of reporter gene expression. Ets-2 transcripts are certainly present in the ovine conceptus (Fig. 1B), and Ets-2 protein is expressed in trophectoderm of bovine conceptuses of equivalent developmental stage [22], but appears to be limiting for driving maximal expression of reporter genes from *IFNT *and *hCG *subunit gene promoters in human choriocarcinoma cells, such as JAr and JEG3, and in 3T3 mouse fibroblasts [23]. Our first goal, therefore, was to determine whether Ets-2 might play a role, possibly a central one, in control of the *Tkdp-1 *gene expression, just as it does in controlling the activities of other signature genes of trophectoderm.

Based on the fact that the *Tkdp-1 *promoter possessed several sites that might bind Ets-2 or one of its relatives, transient transfection experiments were performed in JEG3 choriocarcinoma cells to test the effects of various Ets family transcription factors on the *Tkdp-1 *promoter expression (Fig. 4A). As expected, Ets-2 proved most effective of the factors tested and up-regulated the *Tkdp-1 *promoter about as well as it did the *IFNT *promoter [12]. The ability of Ets-2 to drive expression from the *Tkdp-1 *promoter in 3T3 cells required an activated MAPK pathway (Fig. 5) and was at least partially dependent on the MAPK target residue, Thr 72, in the so-called "pointed" domain of the protein[23]. These results were entirely consistent with Ets-2 effects on the *IFNT *and *hCG *subunit gene promoters when they were examined in 3T3 cells [16,23,28]. In human choriocarcinoma cells, expression was highly responsive to over-expression of the catalytic subunit of PKA. A combination of PKA and Ets-2 over-expression could up-regulate the promoter over 700-fold (Fig. 6A). Again, in all these respects, the *Tkdp-1 *promoter behaved similarly to the *IFNT, hCGα, and hCGβ *promoters [11,16,17,23]. Finally, we have also demonstrated that the Ets-2 effects on the *Tkdp-1 *promoter are silenced by the expression of Oct-4 (Chakrabarty, A. and Roberts, R. M., unpublished results), another feature shared with the, *hCGα*, *hCGβ*, and *IFNT *genes [29,40,41]. These similarities suggest that all these trophoblast-expressed genes share a common transcriptional control mechanism, with a central role played by Ets-2 in each, which allows the genes to be up-regulated soon after the trophectoderm cell lineage first emerges.

In the case of the *IFNT*, *hCGα *and *hCGβ *genes, however, transactivation by Ets-2 is entirely dependent upon the ability of the transcription factor to bind directly to DNA at recognizable, although relatively diverse, Ets-2-binding sequences [11,16,17,23,42]. Strikingly, the proximal/minimal *Tkdp-1 *promoter region between 82 and 140 bp from the *tsp*, which is responsive to Ets-2 (Fig. 2 and 4B), seems physically incapable of forming a stable association with Ets-2 *in vitro *(Fig. 7). The most obvious conclusion is that Ets-2 must form an association with a second transcription factor that binds within this critical region, and, when so positioned, is able to influence transcription from the *Tkdp-1 *gene promoter about as efficiently as it does when it binds directly to the *IFNT *promoter.

The next goal, therefore, was to elucidate the mechanism by which Ets-2 controls *Tkdp-1 *gene expression without a direct interaction with the DNA. Our initial focus was on a conserved CCAAT/enhancer element located between 117 and 109 bp up-stream of the *tsp *(Fig. 3). There are several reasons to consider the possibility that a CCAAT-binding protein might regulate the *Tkdp-1 *gene. First, C/EBP-α, -β and -δ are expressed in human placental cells [29]. Second, C/EBP-β transactivates multiple genes that are expressed by trophoblast cells, including the gene for the homeobox protein distal-less-3 (Dlx3) [43], a transcription factor that binds to the *IFNT *promoter and co-operatively enhances its Ets-2 responsiveness (Ezashi, T. and Roberts, R. M.; unpublished data). Third, recent experiments have suggested a crucial role for C/EBP-α and -β in embryogenesis, since deletion of both genes results in mortality around embryonic day 10–11 due to gross failure in placental development [44]. Fourth, C/EBP-α and -β have been demonstrated to interact with several Ets family members, including Elk-1, Fli-1, Ets-1 [30,32,33,45]. Fifth, C/EBP proteins, particularly C/EBP-β, are responsive to both cAMP/PKA and MAPK pathways [34,46]. Activation of the cAMP/PKA pathway promotes C/EBP-β gene transcription, nuclear translocation, recruitment of transcriptional co-activators (e.g. CBP/p300) and phosphorylation at specific serine residues [46-55], while the major effect of the MAPK pathway on C/EBP-β is mediated through phosphorylation at specific threonine residues, which leads to its activation[55,56].

When over-expressed in JEG-3 cells, none of the C/EBP isoforms -α, -β, and -δ had an effect on the 140 bp *Tkdp-1 *minimal promoter. In presence of Ets-2 (Fig. 8A), a modest increase in promoter activity was observed. When Ets-2+C/EBP-β-transfected cells were treated with 250 μM Br-cAMP, a synergistic increase in promoter activity was observed, particularly in presence of C/EBP-β (Fig. 8B). Thus, in JEG-3 cells, maximal effects of the C/EBP proteins on the *Tkdp-1 *promoter require the participation of both Ets-2 and the cAMP/PKA signaling pathway. Transient transfection experiments and EMSA indicated C/EBP-β was the most potent of the C/EBP isoforms that bound to the CCAAT/enhancer element (Fig. 8A and 8B).

Further importance of the CCAAT/enhancer element in controlling transcriptional activity of the 140 bp *Tkdp-1 *promoter was demonstrated by mutational analysis (Fig. 9A and 9B). Not only was an intact CCAAT element required for C/EBP-β binding and basal promoter activity, but also for Ets-2- and Ets-2+PKA-mediated activation (Fig. 9A). Thus, C/EBP-β bound to the CCAAT/enhancer element is likely to be the target for the cAMP/PKA signaling pathway and also responsible for recruiting Ets-2. Further support for the importance of C/EBP-binding site in Ets-2-dependent activation of the *Tkdp-1 *promoter came from co-transfection experiments in NIH3T3 cells, where the mutated promoter demonstrated markedly reduced Ets-2 and Ets-2+Ras effects (Fig. 9B). Co-IP and pull-down assays confirmed a direct association between C/EBP-β and Ets-2 proteins in JEG-3 cells (Fig. 11A, B and 11C). Together, these experiments indicate that C/EBP-β binds to the *Tkdp-1 *promoter between -117 and -109 and that Ets-2 is recruited to this site via protein-protein interaction with C/EBP-β. It should be noted here that due to the unavailability of an appropriate ovine trophoblast cell line, further demonstration of the *in vivo *association between C/EBP-β and Ets-2 proteins in context of the *ovTkdp-1 *promoter using the chromatin immunoprecipitation assay was not possible.

Our next focus was on a putative AP-1 element at -99 to -93, adjacent to the CCAAT/enhancer motif in the *Tkdp-1 *promoter (Fig. 3), since AP-1 family transcription factors, especially c-Jun and c-Fos are known to interact with both Ets and C/EBP proteins [49,57-61]. As shown by site-directed mutagenesis and transfection experiments, this AP-1 site appeared crucial for maintaining basal activity of the *Tkdp-1 *promoter in JEG-3 cells, as well as for the full PKA responsiveness of the promoter. On the other hand, mutation of the AP-1 site had no effect on Ets-2 responsiveness (Fig. 12A). In other words, PKA-dependent activation of the *Tkdp-1 *promoter may be mediated by some factor bound to the AP-1 site adjacent to the CCAAT/enhancer motif. Pull down experiments with a broad spectrum antibody that recognizes multiple members of the Jun family demonstrated that some of these proteins did indeed bind to this site (Fig. 12C). However, C/EBP-β itself was also a part of the protein complex (Fig. 12B). Since C/EBP proteins bind to DNA as either homo- or heterodimers [62], and C/EBP-β is known to form complexes with c-Jun[63], it is tempting to speculate that the AP-1 site adjacent to the CCAAT element in the *Tkdp-1 *promoter recruits a heterodimer consisting of C/EBP-β and a member of the Jun family of proteins.

A search of the recent draft of the bovine genome sequence (see website) [64] allowed us to identify the putative bovine ortholog of the *Tkdp-1 *gene (between positions 424335-407548 on contig no. NW_928686 on chromosome 13). There is 89% sequence identity between the ovine and bovine genes over the 140 bp region directly upstream of the transcription start site determined for the ovine gene (Additional data file 1 – Fig. 3). The single base mutation in the CCAAT element in the bovine gene (TTAcGCAAT versus TTATGCAAT) would likely not destroy the core C/EBP-binding motif (T*K*NNGNAA*K *; where K = T or G and N = any nucleotide) [65], while the single base mutation in the AP-1-binding site (TGACcCA versus TGACTCA) might well reduce its ability to interact with typical AP-1 proteins. The high degree of conservation in the C/EBP-β binding sequence maintained over the 17 million or so years since the lineage leading to modern day cattle and sheep diverged provides an additional argument for the importance of the CCAAT motif.

## Conclusion

The pattern of expression of the ovine trophoblast Kunitz domain protein-1 produced by the trophectoderm during the peri-implantation stage of embryo development is almost identical to that of IFN-tau and directed by the same transcription factor, Ets-2, in combination with the protein kinase A and mitogen-activated protein kinase signal transduction pathways, but does not involve direct binding of Ets-2 to promoter control elements. Instead, up-regulation of the gene is accomplished indirectly through protein-protein interactions with C/EBP-β. Most importantly, the work reported here demonstrates how Ets-2, a key transcription factor for trophoblast differentiation and function, can control expression of genes having similar spatial and temporal expression patterns via very different mechanisms.

## Methods

### Reverse transcription polymerase chain reaction (RT-PCR) analyses of sheep conceptus RNA

Animal husbandry and surgical procedures were performed according to the protocols approved by the Animal Care and Use Committee at the University of Missouri-Columbia [66, 3097]. Expression patterns of TKDP-1, IFN-τ, Ets-2, C/EBP-β, and ribosomal protein S25 mRNAs were determined by RT-PCR on sheep conceptus/ trophoblast RNA collected on days 14, 15, 16, 17, 19 and 25 of pregnancy. RNA was extracted by using RNA STAT-60 reagent (Tel-Test, Friendswood, TX)[66]. RT reactions were performed on 1–2 μg RNA (pre-treated with deoxyribonuclease; Ambion, Inc., Austin, TX) by using Super-Script III ribonuclease H^- ^reverse transcriptase (Invitrogen, Carlsbad, CA) in presence of 50 μM oligodeoxythymidine (dT) primer. RT reaction product (5 μl) was used for each 50 μl PCR reaction with 100 ng of sense and anti-sense primers (Additional data file 1 – Table 1) and PicoMaxx High Fidelity PCR Master Mix (Stratagene, La Jolla, CA). Twenty-five PCR cycles were used to amplify TKDP-1, IFN-τ and S25 messages, while 30–35 cycles were needed to visualize the Ets-2 and C/EBP-β-mRNAs. PCR products were resolved through 1.5% TAE (Tris-acetate-EDTA)-agarose gels at 100 volts for 1 h and stained with ethidium bromide. In order to confirm that RNA preparations were free of DNA, control RT reactions were performed without initial reverse transcription. As positive controls we employed 10 ng plasmid DNAs encoding the open reading frames for ovTKDP-1, ovIFN-τ4, Ets-2, C/EBP-β and ribosomal protein S25.

### Constructs, antibodies and cell culture reagents

The transcription start point (*tsp*) of the *Tkdp-1 *gene [Genbank:AY940665] is located at ~35 bp downstream of a putative TATA-like sequence (1730 in the *Tkdp-1 *gene) within the 1 Kbp region upstream of the 5^/ ^untranslated region *(UTR) *[2]. Chimeric promoter-reporter plasmids were constructed by amplifying the 1 Kbp region upstream of the 5^/ ^*UTR *by PCR to generate 1000 bp, 558 bp, 352 bp, 254 bp, 192 bp, 140 bp and 82 bp fragments. After verifying their sequences, these products were inserted between the HindIII and XhoI restriction sites on the pGL2 Basic promoter-less plasmid containing the *Luc *reporter gene (Promega, Madison, WI). Additional data file 1 – Table 2 lists all sense primers used in combination with a common anti-sense primer for generating the PCR fragments. Expression plasmids for chicken Ets-1, human Ets-2, mouse Elf-1, human Fli-1, mouse PEA-3, mouse PU.1 were provided by Dr. Michael Ostrowski, Ohio State University, Columbus, OH. The expression vectors for mouse GABP-α and -β1 were from Dr. M. E. Martin, University of Missouri, Columbia, MO. The GABP-α and -β1 plasmids were used together, as GABP functions as a heterodimer. The mutated Ets-2 construct (pCGNEts-2A72) and constitutively active Rasexpression vector pHO6T1 or its parental vector Homer6 have been described elsewhere [2]. The plasmid used to express the constitutively active form of the catalytic subunit of PKA expression construct was a generous gift from Dr. Mark S. Roberson, Cornell University College of Veterinary Medicine, Ithaca, NY. The plasmids for the catalytically inactive PKA expression construct (mutPKA), in which the ATP-binding codon, lysine 72, had been mutated to methionine; [23], and the PKA inhibitor, PKI, [67] were provided by Dr. Richard A. Maurer, University of Iowa, Iowa City, Iowa. Constructs for rat C/EBP-α, mouse C/EBP-β and mouse C/EBP-δ were obtained from Dr. Steven L. McKnight (Southwestern Medical School, Dallas, TX). The *β-galactosidase *gene driven by the Rouse sarcoma virus LTR (pRSVLTR-β-gal) was provided by Dr. R. V. Guntaka, University of Missouri, Columbia, MO. The Renilla-Luciferase reporter plasmid driven by Simian virus 40 enhancer and promoter (pRL-SV40) was purchased from Promega (Madison, WI).

The CCAAT/enhancer element between -117 and -109 and the AP-1 element between -99 and -93 of the *Tkdp-1 *promoter were mutated by using the Quik Change Site-directed Mutagenesis kit from Stratagene (La Jolla, CA). Sequences of the sense and anti-sense primers are listed in additional data file 1 – Table 3. The fidelity of mutation was checked by sequencing with the pGL2 Basic vector-specific GL-1 and GL-2 primers (Promega, Madison, WI).

Affinity purified rabbit polyclonal antibodies for C/EBP-α (*sc-61*), -β (*sc-150*), -δ (*sc-151*), Ets-2 (*sc-*351), pan-Jun (*sc-44*; recognizes c-Jun, Jun B, Jun D, p39), pan-Fos (*sc-413*; recognizes c-Fos, Fos B, Fra-1, Fra-2) and non-immune rabbit IgG (*sc-2027*) were purchased from Santa Cruz Biotechnology, Inc. (Santa Cruz, CA).

Fetal bovine serum was purchased from Harlan (Indianapolis, IN). Media for mammalian cell culture (MEM and DMEM), cell culture-grade sodium pyruvate, L-glutamine and penicillin-streptomycin solutions were purchased from Invitrogen (Carlsbad, CA). Peptidase and phosphatase inhibitors, 8-bromo-cAMP (8-Br-cAMP), and MEM non-essential amino acid solution were purchased from Sigma (Saint Louis, MO).

### Cell culture, transfection and statistical analyses

Transfection experiments were performed in either the human choriocarcinoma JEG-3 cells or the mouse fibroblast NIH3T3 cells (for experiments with activated Ras). Both cell lines were purchased from ATCC (Manassas, VA) and maintained in 5% CO_2 _in air and at 37°C in media (MEM for JEG-3 and DMEM for 3T3 cells) containing 10% heat-inactivated fetal bovine serum and 50 U/ml penicillin and 50 μg/ml streptomycin. Medium for JEG-3 cells was supplemented with 1 mM sodium pyruvate and 1× non-essential amino acid. DMEM for 3T3 cells was supplemented with 4 mM L-glutamine. The day before transfection, cells were plated in 60 mm (JEG-3 cells) or 35 mm dishes (3T3 cells) at a density of 2.5 × 10^5 ^cells/60 mm dish (JEG-3 cells) or 1.7 × 10^5 ^cells/35 mm dish (3T3 cells).

Transient transfection experiments (repeated at least twice in triplicate) in JEG-3 and NIH3T3 cells were performed with calcium phosphate [68] and FuGene 6 reagents (Roche Molecular Biochemicals, Indianapolis, IN), respectively. The pRSVLTR-β-gal (0.025 μg/60 mm dish) and pRL-SV40 (0.008 μg/35 mm dish) constructs were used as internal normalization controls in JEG-3 and 3T3 cells, respectively. Each set of transfections in 60 mm dish was performed with 3.5 μg of *Luc *reporter construct and 0.5 μg of the plasmid DNA for each transcription factor. Cells in each 35 mm dish were transfected with 1.7 μg of *Luc *construct, 0.25 μg Ets-2 or PKA expression construct and 0.6 μg activated Ras expression construct. Total amount of DNA was kept constant in all transfections by including appropriate empty vector. In some experiments, cells were treated with 250 μM 8-Br-cAMP approximately 10 h after transfection. *Luc *and β-galactosidase enzyme assays were usually performed on cell lysates 44–48 h after transfection with commercially available kits (*Luc *and Dual *Luc *Assays system from Promega, Madison, WI and Galactolight Plus systems from Applied Biosystems, Foster City, CA) as 15 sec light outputs from a Turner Designs model 20e luminometer (Sunnyvale, CA).

Transfection results were expressed as average fold induction in *Luc *reporter activity ± SEM. Statistical test on differences in relative *Luc *activities (normalized values) were performed by one way ANOVA followed by Tukey's multiple comparison tests (GraphPad Prism version 4).

### Electrophoretic mobility shift assay (EMSA)

EMSA was carried out with ^32^P-labeled ds Ets-2 oligonucleotides (35 fmole) [69] (Additional data file 1 – Table 4) and 2 μg of ovEts-2-GST fusion protein [21] (with GST as control). Competition was provided by addition of either a 250-fold molar excess of unlabeled Ets-2 consensus oligonucleotide or each of six overlapping oligonucleotides spanning the -150 bp to + 20 bp region on the *Tkdp-1 *promoter (Table 4 in additional data file 1 – data; *tsp *= +1). Competitor oligonucleotides were added prior to the addition of the labeled oligonucleotide to the binding reaction mixtures. Procedures for DNA binding reactions and electrophoresis have been described previously [23]. The specificity of Ets-2 binding was verified by the ability of 0.3 μg of Ets-2 antibody to retard the DNA-protein complex during electrophoresis.

EMSA was also performed with the labeled, ds CCAAT/enhancer element consensus sequence (35 fmol) from the *Tkdp-1 *gene (between -117 and -109) and 10 μg of JEG-3 cell extracts prepared with 200 mM (pH 7.8) sodium phosphate buffer, 0.5% triton X-100, 0.5 mM dithiothreitol and 1× peptidase inhibitor cocktail (Sigma, St. Louis, MO). The specificity of DNA-protein complex formation was verified by adding 250-fold molar excess of unlabeled wild type or mutated C/EBP oligonucleotide (Additional data file 1 – Table 4). In order to identify which of the three C/EBP isoforms among -α, -β and -δ bound to the CCAAT/enhancer element from the *Tkdp-1 *gene, 0.4 μg of affinity purified rabbit polyclonal antibody specific to each these transcription factors was added to each reaction.

### Co-immunoprecipitation reactions (Co-IP)

Rabbit IgG TrueBlot kit (eBioscience, Inc., San Diego, CA) was used forCo-IP experiments with JEG-3 cells treated with 250 μM 8-Br-cAMP for 24 h prior to cell lysis. Cell extracts were prepared with 200 mM (pH 7.8) sodium phosphate buffer, 0.5% triton X-100, 0.5 mM dithiothreitol and 1× each of the peptidase and phosphatase inhibitor cocktails. For immunoprecipitation (IP), 3 μg of each of the rabbit Ets-2 antibody, rabbit C/EBP-β antibody and non-immune rabbit IgG were used per mg of protein in cell extracts. Immunoprecipitated samples were resolved through 10% SDS-PAGE under denaturing conditions and protein bands were transferred onto PVDF membranes (Immobilon-P by Millipore Billerica, MA). Western blotting was performed with a 1:500 dilution of rabbit polyclonal C/EBP-β antibody in blocking solution (25 mM Tris-HCl, pH 7.3, 150 mM NaCl, 0.1% tween-20 and 5% non-fat dry milk). Following incubation with primary antibody, the blot was treated with horseradish peroxidase (HRP) conjugated anti-rabbit IgG (eBioscience, San Diego, CA) at 1:1000 dilution in blocking solution. Membranes were developed with the Phototype-HRP Western Blot Detection System from Cell Signaling Technology, Inc., Beverly, MA, and recorded on Kodak BioMax Light Film (Kodak, Rochester, NY) according to the manufacturers' instructions.

### Pull-down assays

Pull-down assays from JEG-3 cell extracts were performed with biotinylated ds oligonucleotides representing C/EBP and AP-1-binding elements (Additional data file 1 – Table 5). Each pair of sense and anti-sense oligonucleotides was prepared and modified (3^/ ^biotinylation of the sense strand) by MWG-Biotech (High Point, NC). Biotinylated ds oligonucleotide were incubated with 50 μl of immobilized streptavidin (Pierce Biotechnology, Rockford, IL) at 4°C for 1–2 h, followed by addition of 0.5 to 1.0 mg of JEG-3 cell extracts (prepared with 25 mM Tris-HCl, pH 7.5, 150 mM NaCl, 0.5% Triton X-100, 0.5 mM dithiothreitol and 1× each of the peptidase and phosphatase inhibitor cocktails (Sigma, Saint Louis, MO) at 4°C for overnight with gentle mixing. The immobilized DNA-protein complex was washed (3×) with cold 25 mM Tris-HCl, pH 7.5, 150 mM NaCl and boiled with 5× SDS-loading dye under denaturing condition and resolved through 10% SDS-PAGE gels. Proteins were transferred from the gel to the PVDF membrane as described above. Western blotting was performed with 1:750 dilution of each of the rabbit polyclonal Ets-2, C/EBP-β, pan-Jun and pan-Fos antibodies and developed by the Western-Star Systems from Applied Biosystems (Bedford, MA) according to the manufacturer's instruction.

## Supplementary Material

Additional data file 1 The additional data file 1 consists of tables of sense and anti-sense primers used for RT-PCR amplification of ovTKDP-1, IFN-τ, ribosomal protein S25, Ets-2 and C/EBP-β messages, oligonucleotides for PCR-cloning of the *ovTkdp-1 *promoter constructs, site-directed mutagenesis of the C/EBP and AP-1 sites in the 140 bp *ovTkdp-1 *promoter, sense oligonucleotides used for EMSA and sense and anti-sense oligonucleotides used for biotinylated pull-down assays. It also contains figures of putative Ets-binding sites in the *ovTkdp-1 *promoter, effect of 8-Br-cAMP on the Ets-2-mediated activation of the 1000 bp *ovTkdp-1 *promoter-*Luc *reporter construct and comparison of the *ovTkdp-1 *minimal promoter with its putative bovine ortholog. Click here for file
