# Supplementary material for: Ets-2 and C/EBP-beta are important mediators of ovine trophoblast Kunitz domain protein-1 gene expression in trophoblast
Source: BMC Mol Biol. 2007 Feb 27;8:14. doi: 10.1186/1471-2199-8-14 (PMC1817651; doi:10.1186/1471-2199-8-14)
Supplement: Additional data file 1 — The additional data file 1 consists of tables of sense and anti-sense primers used for RT-PCR amplification of ovTKDP-1, IFN-τ, ribosomal protein S25, Ets-2 and C/EBP-β messages, oligonucleotides for PCR-cloning of the ovTkdp-1 promoter constructs, site-directed mutagenesis of the C/EBP and AP-1 sites in the 140 bp ovTkdp-1 promoter, sense oligonucleotides used for EMSA and sense and anti-sense oligonucleotides used for biotinylated pull-down assays. It also contains figures of putative Ets-binding sites in the ovTkdp-1 promoter, effect of 8-Br-cAMP on the Ets-2-mediated activation of the 1000 bp ovTkdp-1 promoter-Luc reporter construct and comparison of the ovTkdp-1 minimal promoter with its putative bovine ortholog. [file 1471-2199-8-14-S1.doc]

**Additional Data File**

**Ets-2 and C/EBP-beta are important mediators of ovine trophoblast Kunitz domain protein-1 gene expression in trophoblast**

**Anindita Chakrabarty^1,§^ and R. Michael. Roberts^2,3^,***

**Contents:**

Table 1: Sense and anti-sense primers used for RT-PCR amplification of

ovTKDP-1, IFN-τ, ribosomal protein S25, Ets-2 and C/EBP-β messages.

Table 2: Sense and anti-sense primers used for PCR-cloning of the

*ovTkdp-1* promoter constructs.

Table 3: Sense and anti-sense primers used for site-directed mutagenesis of the C/EBP and AP-1 sites in the 140 bp *ovTkdp-1* promoter.

Table 4: Sense Oligonucleotides used for EMSA.

Table 5: Sense and anti-sense oligonucleotides used for biotinylated pull-down assays for C/EBP-β and AP-1

Figure 1: Putative Ets-binding sites in the *ovTkdp-1* promoter.

Figure 2: Effect of different concentrations of 8-Br-cAMP (50, 250, 500 and 1000 μM) on the Ets-2-mediated activation of the 1000 bp *ovTkdp-1* promoter-*Luc* reporter construct in JEG-3 cells.

Figure 3: Comparison of the *ovTkdp-1* minimal promoter with its putative bovine ortholog.

Table 1: Sense and anti-sense primers used for RT-PCR amplification of

ovTKDP-1, IFN-τ, ribosomal protein S25, Ets-2 and C/EBP-β messages

**Primers Sequence**

ovTKDP-1 Sense 5/-ATTGGAGCCCTCCTGCAAG-3/

ovTKDP-1 Anti-sense 5/-GCATTGCAGTCACCTGTT-3/

IFN-τ Sense 5/-TTACCTATCTCAGAGACTCA-3/

IFN-τ Anti-sense 5/-AGTCATCAAGGTGAGTTCAG-3/

S25 Sense 5/-CCCAAGGACGACAAG-3/

S25 Anti-sense 5/-TCATGCATCTTCACCAGCAGC-3/

Ets-2 Sense 5/-GAAGATCAGTATGAAGAAAATTCGCA-3/

Ets-2 Anti-sense 5/-AATGACTGGCAGGACTTGTCG-3/

C/EBP Sense 5/- AAGCCGCGACAAGGCC-3/

C/EBP Anti-sense 5/-CAGCTGCTTGAACAAGTT-3/

Table 2: Sense and anti-sense primers used for PCR-cloning of the

*ovTkdp-1* promoter constructs

**Primers Sequence**

1000 Sense 5/-GCCTGAGAAGTTTACTCA-3/

558 Sense 5/-CAACATCACCTTCACCAG-3/

352 Sense 5/-GCTGGTTACTGATCTGCC-3/

254 Sense 5/-CCATATAGGCCACACTCC-3/

192 Sense 5/-CATCAAGTGATAAGTGCTC-3/

140 Sense 5/-GGAGGCATGGGTAGGTAG-3/

82 Sense 5/-TTGCCCACTCCCCTCCTTATC-3/

Anti-sense 5/-GTGGCCTTGCAGGAGGGC-3/

Table 3: Sense and anti-sense primers used for site-directed mutagenesis of the C/EBP and AP-1 sites in the 140 bp *ovTkdp-1* promoter

**Primers Sequence**

C/EBP Sense 5/GGCATGGGTAGGTAGGGAGGTTATcCcccAAACAGGACTGAC-3/

C/EBP Anti-sense 5/-GTCAGTCCTGTTTgggGgATAACCTCCCTACCTACCCATGCC-3/

AP-1 Sense 5/-GCAATAAACAGGACgGACTtAACTCCTCCCCTTGCC-3/

AP-1 Anti-sense 5/-GGCAAGGGGAGTTaAGTCcGTCCTGTTTATTGC-3/

Table 4: Sense Oligonucleotides used for EMSA

**Oligonucleotide Sequence**

TKDP-1/1 5/-ACAGATGCAGGAGGCATGGG -3/

TKDP-1/2 5/CATGGGTAGGTAGGGAGGTTATGCAATAAACAGGACTGACTCAA

CTCCTCC-3/

TKDP-1/3 5/-TCCTCCCCTTGCCCACTCCCCTCCTTATCACCTGATCTCACGACTCA-3/

TKDP-1/4 5/-CTGATCTCACGACTCAGCCAGAATATTAAAAGCTC-3/

TKDP-1/5 5/-CAGTGTGTCCATCCTCAGCATCCTCAGGAGCCCTC-3/

TKDP-1/6 5/-AGGAGCCCTCCTGCAAGGCC-3/

Ets-2 consensus 5/-CTAGGACCGGAAGTGGGAGT-3/

wt C/EBP 5/-AGGTAGGGAGGTTATGCAATAAACAGGACT-3/

mut C/EBP 5/-AGGTAGGGAGGTTATcCcccAAACAGGACT-3/

Table 5: Sense and anti-sense oligonucleotides used for biotinylated pull-down assays for C/EBP-β and AP-1

**Primers Sequence**

C/EBP Sense 5/-AGGTAGGGAGGTTATGCAATAAACAGGACT-3/

C/EBP Anti-sense 5/-AGTCCTGTTTATTGCATAACCTCCCTACCT-3/

AP-1 Sense 5/-TAAACAGGACTGACTCAACTCCTCCCC-3/

AP-1 Anti-sense 5/-GGGGAGGAGTTGAGTCAGTCCTGTTTA-3/

T GCCTGGAGAA GTTACTCATT ACTGTTTTTA TTACTACTAG -960

GAAGGATTTG CCTTCTAGGG AGGAGGAACA TCTGCCTGTT GCATCCATTG CTTCCATGGC -900

Ets Ets-like

AACCACATGG GTACAGAGCA TGTGTCTCAT GTGACTCCTG AAGACACGGG TTCTGAGAGG -840

TTTAAATCCT CAGGCAAAAA GGTAGGACAA TGCAGAGCTG GTACCCAAAC TGAGTCCTAT -780

GGCCCCATCG ACAGAGAGGG AGAGAGCAGG AGGAAGTGGG TGGCTATGGT GTTCTGAGGT -720

Ets-like

CTCCTGCTGT CCACTCAGGG AGACTGACTG CTCTCCTGTT GGTGAAACCA GGTGAGGGGT -660

GACTGCTGGA CATGCTGTGG AGACAGATTC TGCTCCCTGT TTCCCCTCAG CCACCCTCCA -600

TTACTCATTG TTTGATACAT AAAGACGGTA ATTGCCCAGG ACAACATCAC CTTCACCAGC -540

TAAGCCCCCT TCCTGGACCA AGGTCTGGAC AAGTGCAAGT GTCCATCCAC CAGAGAAGCT -480

GCGGCAACGG CTCGAGAAGG TGACCTGTGT CAGTTGCCAT CTGAGGTAGG GGAGGGGGGT -420

CAGGACACTG AGGTCCAGGT GAGACGACAC CTGCAGGAGG CTGGAATGGA GGACCTGGTT -360

Ets-like

ACCGCACCGC TGGTTACTGA TCTGCCCTCG GATCTGCCCT CATCCACCTA CTTATCGTTC -300

Ets

ATCACATGCC AGGCACTGCT CTAGGGGCTG GATGCTCCCT GACTATCCAT ATAGGACACA -240

Ets

CTCCATCTCT CTGATCACAA TCTGTGTTTA CACAAGACCC ACAGGCATCA TCAAGTGATA -180

AGTGCTCTTA CTGGCCTTTT GAGCCTGCAG ACAGATGCAG GAGGCATGGG TAGGTAGGGA -120

Ets-like Ets-like

GGTTATGCAA TAAACAGGAC TGACTCAACT CCTCCCCTTG CCCACTCCCC TCCTTATCAC -60

+1

CTGATCTCAC GACTCAGCCA GAA**TATTAA**A AGCTCAGTGT GTCCATCCTC AGCATCCTC**A +1**

TATA-like Sequence Ets Ets

GGAGCCCTCC TGCAAGCCAC CGAG**ATG**AGA +31

Ets-like Translation Initiation Site

**Figure 1**

**Putative Ets-binding sites in the *ovTkdp-1* promoter.** Ets core motifs, putative TATA box, transcription and translation initiation sites are underlined and marked. Putative Ets-binding sites included in the 140 bp minimal promoter are highlighted.

**Figure 2**

**Effect of different concentrations of 8-Br-cAMP (50, 250, 500 and 1000 μM) on the Ets-2-mediated activation of the 1000 bp *ovTkdp-1* promoter-*Luc* reporter construct in JEG-3 cells.** *Luc* activities are expressed relative to that of the basal activity of the 1000 bp construct and data are shown as mean ± SEM (n=3).

***boTkdp-1*** GGAGGCATGGTTAGGTAGGGAGATTAcGCAATAAACAGGACTGACcCAACTCCTCCCCTT

***ovTkdp-1*** GGAGGCATGGGTAGGTAGGGAGGTTATGCAATAAACAGGACTGACTCAACTCCTCCCCTT

**C/EBP AP-1**

***boTkdp-1*** GCCCACTCCCCTCCTCATCACCTGATCCCACCCCTCAGCCAGGATATTAAA-GCTCAGTG

***ovTkdp-1*** GCCCACTCCCCTCCTTATCACCTGATCTCACGACTCAGCCAGAATATTAAAAGCTCAGTG

**TATA-like**

***boTkdp-1*** ----CATCCCCAGCATCCTC**A**

***ovTkdp-1*** TGTCCATCCTCAGCATCCTC**A**

**+1**

**Figure 3**

**Comparison of the *ovTkdp-1* minimal promoter with its putative bovine ortholog.** First 140 bp upstream of the transcription start point (+1) are shown. C/EBP, AP-1 and TATA-like elements are highlighted. Transcription initiation site is marked as +1 in bold letter. Mismatched nucleotides within the *boTkdp-1* gene for the putative C/EBP and AP-1 sites are shown in lower cases.
